# 2-Cyano-*N*′-[1-(pyridin-2-yl)ethyl­idene]acetohydrazide

**DOI:** 10.1107/S1600536812042869

**Published:** 2012-10-24

**Authors:** Xiao-Yi Zhang, Xiao-Lin Han, Zhi-Bin Qian

**Affiliations:** a School of Pharmacy, Xinxiang Medical University, Xinxiang Henan 453003, People’s Republic of China; b The Hematology Department of the First Affiliated Hospital of Xinxiang Medical University, Weihui Henan 453100, People’s Republic of China; c School of Basic Medical Sciences, Xinxiang Medical University, Xinxiang Henan 453003, People’s Republic of China

## Abstract

In the title compound, C_10_H_10_N_4_O, the dihedral angle between the pyridine ring and the –C=O(CH_2_)CN group is 24.08 (12)°. In the crystal, inversion dimers linked by pairs of N—H⋯N hydrogen bonds generate *R*_2_^2^(8) loops.

## Related literature

For the biological activity of hydrazone compounds, see: Rauf *et al.* (2008 ▶); Zhang *et al.* (2012 ▶). For related structures, see: Taha *et al.* (2012 ▶); Kargar *et al.* (2012 ▶); Rassem *et al.* (2012 ▶).
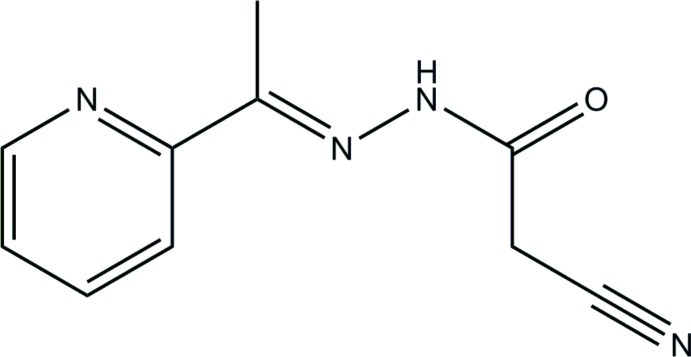


## Experimental

### 

#### Crystal data

C_10_H_10_N_4_O
*M* *_r_* = 202.22
Monoclinic, 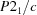

*a* = 8.192 (2) Å
*b* = 14.520 (2) Å
*c* = 8.7340 (17) Å
β = 98.466 (2)°
*V* = 1027.6 (4) Å^3^
*Z* = 4
Mo *K*α radiation
μ = 0.09 mm^−1^
*T* = 298 K
0.17 × 0.13 × 0.12 mm

#### Data collection

Bruker SMART CCD diffractometer
Absorption correction: multi-scan (*SADABS*; Sheldrick, 1996) ▶ *T*_min_ = 0.985, *T*_max_ = 0.989
6189 measured reflections
2222 independent reflections
1128 reflections with *I* > 2σ(*I*)
*R*_int_ = 0.040

#### Refinement

*R*[*F* ^2^ > 2σ(*F* ^2^)] = 0.064
*wR*(*F* ^2^) = 0.143
*S* = 1.03
2222 reflections
140 parameters
1 restraint
H atoms treated by a mixture of independent and constrained refinement
Δρ_max_ = 0.14 e Å^−3^
Δρ_min_ = −0.18 e Å^−3^

### 

Data collection: *SMART* (Bruker, 1998 ▶); cell refinement: *SAINT* (Bruker, 1998 ▶); data reduction: *SAINT*; program(s) used to solve structure: *SHELXS97* (Sheldrick, 2008 ▶); program(s) used to refine structure: *SHELXL97* (Sheldrick, 2008 ▶); molecular graphics: *SHELXTL* (Sheldrick, 2008 ▶); software used to prepare material for publication: *SHELXTL*.

## Supplementary Material

Crystal structure: contains datablock(s) global, I. DOI: 10.1107/S1600536812042869/hb6969sup1.cif

Structure factors: contains datablock(s) I. DOI: 10.1107/S1600536812042869/hb6969Isup2.hkl

Supplementary material file. DOI: 10.1107/S1600536812042869/hb6969Isup3.cml

Additional supplementary materials:  crystallographic information; 3D view; checkCIF report

## Figures and Tables

**Table 1:** Hydrogen-bond geometry (Å, °)

*D*—H⋯*A*	*D*—H	H⋯*A*	*D*⋯*A*	*D*—H⋯*A*
N3—H3*A*⋯O1^i^	0.90 (1)	2.05 (1)	2.929 (2)	167 (2)

Symmetry code: (i) 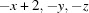.

**Fig. 1.**
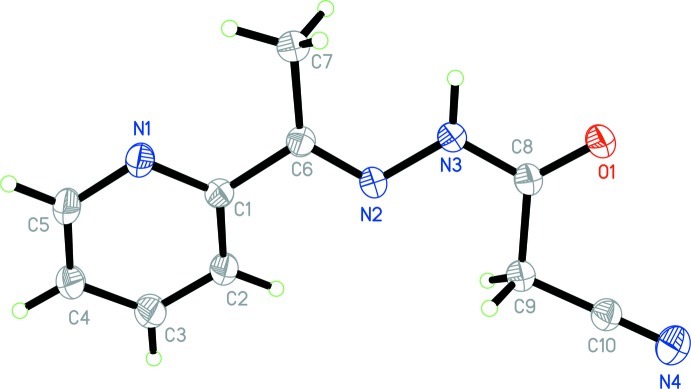
Molecular structure of the title compound with 30% thermal ellipsoids.

**Fig. 2.**
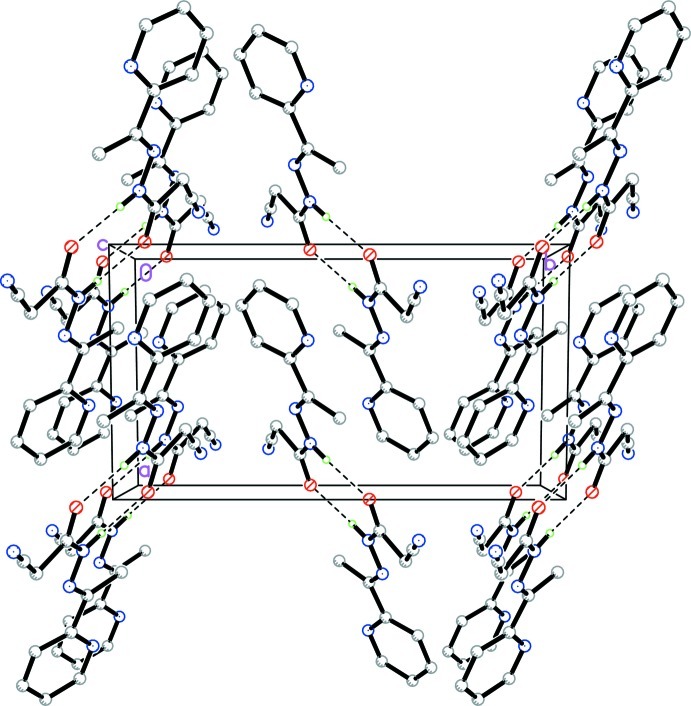
Molecular packing diagram of the title compound, viewed down the c axis. Hydrogen bonds are drawn as dashed lines.

## supplementary crystallographic information

### Comment

Hydrazone compounds bearing biological active functional groups -C(O)-NH-N=CH- are readily prepared by the condensation reactions of hydrazines with various aldehydes (e.g. Rauf *et al.*, 2008; Zhang *et al.*, 2012). In the present work, the title new hydrazone compound, derived from 2-acetylpyridine and cyanoacetohydrazide, is reported.

The molecule of the compound adopts a *trans* conformation about the C6=N2 double bond (Fig. 1). The torsion angles of C6-N2-N3-C8, N2-N3-C8-C9, and N3-C8-C9-C10 are 4.8 (3), 5.1 (3), and 6.5 (3)°, respectively. The bond lengths are comparable to those in similar compounds (Taha *et al.*, 2012; Kargar *et al.*, 2012; Rassem *et al.*, 2012). The crystal structure of the compound features N—H···O hydrogen bonds (Table 1), generating dimers (Fig. 2).

### Experimental

2-Acetylpyridine (1.0 mmol, 0.121 g) and cyanoacetohydrazide (1.0 mmol, 0.991 g) were mixed and stirred in methanol (50 mL) at room temperature for 1 h. Colorless block-shaped single crystals were obtained after slow evaporation of the solution in air for a few days.

### Refinement

H3A attached to N3 was located in a difference Fourier map and was refined isotropically, with N—H distance of 0.90 (1) Å. The remaining hydrogen atoms were positioned geometrically and allowed to ride on their parent atoms, with C—H = 0.93–0.97 Å for aromatic and CH_2_ and 0.96 Å for CH_3_. The U_iso_ values were constrained to be 1.5U_eq_ of the carrier atom for methyl and 1.2U_eq_ for the remaining H atoms. A rotating group model was used for the methyl group.

### Crystal data

**Table:**

C_10_H_10_N_4_O	*F*(000) = 424
*M**_r_* = 202.22	*D*_x_ = 1.307 Mg m^−^^3^
Monoclinic, *P*2_1_/*c*	Mo *K*α radiation, λ = 0.71073 Å
*a* = 8.192 (2) Å	Cell parameters from 1009 reflections
*b* = 14.520 (2) Å	θ = 2.7–24.5°
*c* = 8.7340 (17) Å	µ = 0.09 mm^−^^1^
β = 98.466 (2)°	*T* = 298 K
*V* = 1027.6 (4) Å^3^	Block, colorless
*Z* = 4	0.17 × 0.13 × 0.12 mm

### Data collection

**Table:**

Bruker SMART CCD diffractometer	2222 independent reflections
Radiation source: fine-focus sealed tube	1128 reflections with *I* > 2σ(*I*)
Graphite monochromator	*R*_int_ = 0.040
ω scans	θ_max_ = 27.0°, θ_min_ = 2.7°
Absorption correction: multi-scan (*SADABS*; Sheldrick, 1996)	*h* = −9→10
*T*_min_ = 0.985, *T*_max_ = 0.989	*k* = −18→12
6189 measured reflections	*l* = −11→11

### Refinement

**Table:**

Refinement on *F*^2^	Primary atom site location: structure-invariant direct methods
Least-squares matrix: full	Secondary atom site location: difference Fourier map
*R*[*F*^2^ > 2σ(*F*^2^)] = 0.064	Hydrogen site location: inferred from neighbouring sites
*wR*(*F*^2^) = 0.143	H atoms treated by a mixture of independent and constrained refinement
*S* = 1.03	*w* = 1/[σ^2^(*F*_o_^2^) + (0.0562*P*)^2^ + 0.0523*P*] where *P* = (*F*_o_^2^ + 2*F*_c_^2^)/3
2222 reflections	(Δ/σ)_max_ < 0.001
140 parameters	Δρ_max_ = 0.14 e Å^−^^3^
1 restraint	Δρ_min_ = −0.18 e Å^−^^3^

### Special details

**Table:**

Geometry. All esds (except the esd in the dihedral angle between two l.s. planes) are estimated using the full covariance matrix. The cell esds are taken into account individually in the estimation of esds in distances, angles and torsion angles; correlations between esds in cell parameters are only used when they are defined by crystal symmetry. An approximate (isotropic) treatment of cell esds is used for estimating esds involving l.s. planes.
Refinement. Refinement of F^2^ against ALL reflections. The weighted R-factor wR and goodness of fit S are based on F^2^, conventional R-factors R are based on F, with F set to zero for negative F^2^. The threshold expression of F^2^ > 2sigma(F^2^) is used only for calculating R-factors(gt) etc. and is not relevant to the choice of reflections for refinement. R-factors based on F^2^ are statistically about twice as large as those based on F, and R- factors based on ALL data will be even larger.

### Fractional atomic coordinates and isotropic or equivalent isotropic displacement parameters (Å^2^)

**Table:**

	*x*	*y*	*z*	*U*_iso_*/*U*_eq_	
N1	0.3684 (2)	0.07074 (13)	0.3032 (2)	0.0623 (6)	
N2	0.6478 (2)	0.10204 (13)	0.0382 (2)	0.0537 (5)	
N3	0.7946 (2)	0.06818 (13)	0.0033 (2)	0.0583 (5)	
N4	0.8363 (3)	0.17583 (17)	−0.4976 (3)	0.0901 (8)	
O1	0.97937 (19)	0.06914 (12)	−0.16256 (18)	0.0727 (5)	
C1	0.4400 (3)	0.11185 (15)	0.1938 (2)	0.0504 (6)	
C2	0.3705 (3)	0.18833 (17)	0.1157 (3)	0.0667 (7)	
H2	0.4223	0.2167	0.0404	0.080*	
C3	0.2244 (3)	0.22169 (19)	0.1506 (3)	0.0805 (9)	
H3	0.1764	0.2733	0.0993	0.097*	
C4	0.1494 (3)	0.17926 (19)	0.2605 (3)	0.0714 (7)	
H4	0.0494	0.2006	0.2851	0.086*	
C5	0.2256 (3)	0.10466 (18)	0.3330 (3)	0.0686 (7)	
H5	0.1747	0.0755	0.4082	0.082*	
C6	0.5976 (3)	0.07141 (15)	0.1606 (2)	0.0512 (6)	
C7	0.6822 (3)	−0.00024 (17)	0.2651 (3)	0.0675 (7)	
H7A	0.6830	−0.0574	0.2098	0.101*	
H7B	0.6246	−0.0083	0.3522	0.101*	
H7C	0.7936	0.0187	0.3006	0.101*	
C8	0.8458 (3)	0.09339 (15)	−0.1295 (3)	0.0554 (6)	
C9	0.7285 (3)	0.15165 (16)	−0.2381 (2)	0.0587 (6)	
H9A	0.6215	0.1218	−0.2570	0.070*	
H9B	0.7146	0.2109	−0.1904	0.070*	
C10	0.7896 (3)	0.16532 (16)	−0.3836 (3)	0.0594 (6)	
H3A	0.860 (2)	0.0304 (13)	0.066 (2)	0.080*	

### Atomic displacement parameters (Å^2^)

**Table:**

	*U*^11^	*U*^22^	*U*^33^	*U*^12^	*U*^13^	*U*^23^
N1	0.0625 (14)	0.0709 (13)	0.0578 (12)	0.0094 (11)	0.0226 (10)	0.0093 (10)
N2	0.0449 (11)	0.0645 (13)	0.0536 (11)	0.0050 (9)	0.0137 (9)	0.0024 (9)
N3	0.0490 (13)	0.0724 (14)	0.0548 (12)	0.0110 (10)	0.0125 (9)	0.0116 (10)
N4	0.0921 (18)	0.1099 (19)	0.0729 (15)	0.0051 (14)	0.0278 (13)	0.0163 (14)
O1	0.0543 (11)	0.0962 (13)	0.0717 (11)	0.0180 (9)	0.0226 (9)	0.0185 (9)
C1	0.0485 (14)	0.0581 (14)	0.0448 (12)	−0.0002 (12)	0.0069 (10)	−0.0003 (11)
C2	0.0622 (17)	0.0784 (18)	0.0631 (15)	0.0105 (14)	0.0215 (13)	0.0169 (13)
C3	0.0741 (19)	0.091 (2)	0.0809 (19)	0.0283 (16)	0.0261 (16)	0.0236 (16)
C4	0.0623 (17)	0.0910 (19)	0.0649 (16)	0.0209 (15)	0.0232 (14)	0.0064 (15)
C5	0.0664 (17)	0.0843 (19)	0.0610 (15)	0.0076 (15)	0.0291 (13)	0.0074 (14)
C6	0.0486 (14)	0.0581 (14)	0.0471 (12)	−0.0015 (11)	0.0081 (11)	0.0011 (11)
C7	0.0607 (17)	0.0816 (17)	0.0616 (15)	0.0133 (14)	0.0131 (12)	0.0181 (13)
C8	0.0481 (15)	0.0633 (16)	0.0568 (14)	0.0015 (12)	0.0143 (12)	0.0027 (12)
C9	0.0536 (15)	0.0676 (16)	0.0564 (13)	0.0062 (12)	0.0132 (11)	0.0058 (12)
C10	0.0594 (16)	0.0618 (15)	0.0583 (14)	0.0026 (12)	0.0131 (13)	0.0045 (12)

### Geometric parameters (Å, º)

**Table:**

N1—C5	1.331 (3)	C3—H3	0.9300
N1—C1	1.333 (2)	C4—C5	1.359 (3)
N2—C6	1.280 (2)	C4—H4	0.9300
N2—N3	1.374 (2)	C5—H5	0.9300
N3—C8	1.341 (3)	C6—C7	1.486 (3)
N3—H3A	0.899 (10)	C7—H7A	0.9600
N4—C10	1.128 (3)	C7—H7B	0.9600
O1—C8	1.224 (2)	C7—H7C	0.9600
C1—C2	1.382 (3)	C8—C9	1.506 (3)
C1—C6	1.486 (3)	C9—C10	1.446 (3)
C2—C3	1.366 (3)	C9—H9A	0.9700
C2—H2	0.9300	C9—H9B	0.9700
C3—C4	1.361 (3)		
			
C5—N1—C1	117.8 (2)	N2—C6—C1	114.9 (2)
C6—N2—N3	117.41 (19)	N2—C6—C7	125.31 (19)
C8—N3—N2	119.3 (2)	C1—C6—C7	119.78 (18)
C8—N3—H3A	117.5 (15)	C6—C7—H7A	109.5
N2—N3—H3A	123.2 (15)	C6—C7—H7B	109.5
N1—C1—C2	121.4 (2)	H7A—C7—H7B	109.5
N1—C1—C6	116.7 (2)	C6—C7—H7C	109.5
C2—C1—C6	121.88 (19)	H7A—C7—H7C	109.5
C3—C2—C1	119.1 (2)	H7B—C7—H7C	109.5
C3—C2—H2	120.5	O1—C8—N3	122.0 (2)
C1—C2—H2	120.5	O1—C8—C9	121.5 (2)
C4—C3—C2	119.9 (2)	N3—C8—C9	116.48 (19)
C4—C3—H3	120.1	C10—C9—C8	111.03 (18)
C2—C3—H3	120.1	C10—C9—H9A	109.4
C5—C4—C3	117.7 (2)	C8—C9—H9A	109.4
C5—C4—H4	121.1	C10—C9—H9B	109.4
C3—C4—H4	121.1	C8—C9—H9B	109.4
N1—C5—C4	124.1 (2)	H9A—C9—H9B	108.0
N1—C5—H5	118.0	N4—C10—C9	179.6 (3)
C4—C5—H5	118.0		

### Hydrogen-bond geometry (Å, º)

**Table:**

*D*—H···*A*	*D*—H	H···*A*	*D*···*A*	*D*—H···*A*
N3—H3*A*···O1^i^	0.90 (1)	2.05 (1)	2.929 (2)	167 (2)

Symmetry code: (i) −*x*+2, −*y*, −*z*.
